# In this issue of *Current Oncology*

**Published:** 2008-01

**Authors:** M. McLean

The year 2008 will see the continued expansion of *Current Oncology,* Canada’s only multidisciplinary journal devoted to cancer. In that spirit, the supplement “Going Beyond Efficacy,” edited by Dr. Sunil Verma, is being published together with this first issue of main journal. Dr. Verma asked leaders from across the country to focus on some serious issues being faced by cancer patients and their health care providers. The result is an eclectic mix of topics, including management of nausea and vomiting, neutropenia and anemia, thromboembolism, and iatrogenic osteopenia; use of bisphosphonates and concerns regarding bisphosphonate-related osteonecrosis; and approaches to bone metastasis and palliation. The supplement is not “bagged” with the current issue, but will be published simultaneously online at the *Current Oncology* Web site (www.current-oncology.com). I am also very pleased to add that Dr. Verma has accepted an invitation the join the editorial board of the journal, co-editing the Medical Oncology section, and that Dr. Raymond Nagle has also agreed to join us and to edit a new section on Oncologic Pathology. Also joining us, I am very pleased to report, are Drs. Torgny Stigbrand (Biomarkers in Oncology) from Umeå, Sweden, Herbert Nieburgs (Oncologic History) from Worcester, Massachusetts, and Victor Ling (Translational Research) from Vancouver, British Columbia.

In the issue now in your hands, I am delighted to include an Updates and Developments in Oncology guest editorial by Drs. Serge Hardy and Michel Tremblay, discussing the “changing” role of protein tyrosine phosphatases. In addition, I note that Dr. Tremblay has graciously accepted an invitation to become involved on our editorial board, which has continued to increase in breadth and calibre throughout 2007.

Drs. Stephen Sagar and Raimond Wong have written the first of a two-part series on Chinese medicine and biomodulation in cancer patients, the second part of which is scheduled for the second issue of 2008. Our guidelines series includes recommendations on supportive care of neutropenia by Dr. Tom Kouroukis and colleagues (a topic therefore deliberately not covered in “Going Beyond Efficacy”) and cardiac management during adjuvant trastuzumab therapy from the Canadian Trastuzumab Working Group. Case report topics include radiation recall dermatitis and (illegal) drug-induced “crack lung and heart.” Dr. Nancy Levesque and colleagues discuss the use of high-dose interferon alfa-2b in melanoma, and writer Maya Chaddah reports on the effective—and expanding—role of the Ontario Cancer Research Ethics Board.

In regard to future articles, I urge all authors considering online submission to carefully review the changing requirements for such submissions. Modifications have been made to reflect the needs of our indexing agencies and the recent policy changes concerning conflict of interest, informed consent, and human and animal rights published last year by the International Committee of Medical Journal Editors.

The stated aim of *Current Oncology* is that it remain complimentary for our readership, a goal that will likely continue for the foreseeable future. The costs of publication are, in large part, borne by advertising revenues and unrestricted educational grants, mainly from the pharmaceutical industry. I have never felt that this practice has caused a conflict of interest, given the high calibre of our editorial writers and the total acceptance of this position by our colleagues in industry. In the present issue, we are embarking on a new concept with the appearance of specific information updates again sponsored by unrestricted grants and written by experts in their respective fields, at the editorial board’s specific invitation. We see these “updates” as a fast track to publication, but one nonetheless subject to independent peer review. Our thanks to Drs. Sunil Verma and Anil Joy for stepping up and testing out this concept for us.

Lastly, a happy and productive 2008 to all of you from all of us at *Current Oncology,* Canada’s multi-disciplinary cancer journal, as we enter our 15th year of publication and approach our 300th published manuscript.

